# Semen parameters in men with varicocele: DNA fragmentation, chromatin packaging, mitochondrial membrane potential, and apoptosis

**DOI:** 10.5935/1518-0557.20170053

**Published:** 2017

**Authors:** Felipe Dieamant, Claudia G. Petersen, Ana L. Mauri, Vanessa Conmar, Mariana Mattila, Laura D. Vagnini, Adriana Renzi, Bruna P Costa, Camila Zamara, João Batista A. Oliveira, Ricardo L.R. Baruffi, José G. Franco Jr.

**Affiliations:** 1 Center for Human Reproduction Prof Franco Jr. - Ribeirão Preto, Brazil.; 2 Paulista Center for Diagnosis, Research and Training - Ribeirão Preto, Brazil.

**Keywords:** varicocele, DNA damage, DNA fragmentation, chromatin packaging, inactive mitochondria, apoptosis

## Abstract

**Objective:**

To evaluate varicocele effects on conventional semen parameters: sperm DNA fragmentation, chromatin packaging, mitochondrial membrane potential (MMP) and sperm apoptosis.

**Methods:**

A cross-sectional study was carried out with semen samples from 2,399 men of couples who attended an infertility clinic. A total of 16.3% (391/2399) of the men were diagnosed with varicocele by a urologist.

**Results:**

A regression analysis revealed that the percentages of sperm with DNA fragmentation, abnormal chromatin packaging, and abnormal MMP were significantly increased in individuals with varicocele, when compared to men without varicocele. Apoptosis was not influenced by varicocele. Conventional semen parameters were significantly worse in individuals with the disease. On the other hand, in men with varicocele, Spearman's correlation demonstrated that early apoptosis and abnormal MMP showed a positive and significant correlation with sperm DNA fragmentation.

**Conclusion:**

Men with varicocele had worse semen parameters, including increased levels of sperm DNA fragmentation, inactive mitochondria, and abnormal chromatin packaging. These changes are possible causes of infertility in individuals with varicocele.

## INTRODUCTION

Since 1955, there has been evidence that varicocele, defined as an abnormal dilatation of testicular veins in the pampiniform plexus and by retrograde blood flow in the internal spermatic veins because of incompetent or absent valves, has been associated with decreased sperm quality and thus, male infertility (Tulloch *et al*., 1955; Gat *et al*., 2004). This pathology is considered one of the main causes of male infertility; approximately 15% of the normal male population, and up to 40% of infertile men are believed to have clinical or subclinical varicocele (Witt & Lipshultz, 1993; Pastuszak & Wang, 2015).

Patients with varicocele present with abnormal spermatogenesis, but the exact pathophysiological mechanism by which varicocele promotes male-factor infertility still remains uncertain, although several possible causes have been proposed including testicular hypoxia due to venous stasis, hormonal dysfunction, reflux of renal and adrenal toxic metabolites, hypertension in the internal spermatic veins, and higher testicular temperature (Naughton *et al*., 2001; Saleh *et al*., 2003; Baccetti *et al*., 2006). Furthermore, high levels of oxidative stress, which arises from an unbalance between the over-production of reactive oxygen species (ROS) and the reduced total antioxidant capacity, which could result in alterations of semen parameters and sperm DNA integrity, have been observed in men with varicocele (Hendin *et al*., 1999; Smith *et al*., 2006).

High levels of sperm with DNA damage have been reported in the ejaculate of patients with varicocele compared to fertile men (Saleh *et al*., 2003; Chen *et al*., 2004). Plausible causes of sperm DNA fragmentation include increased levels of ROS, smoking, drug use, advanced age, chemotherapy or radiotherapy, and infections (Simşek *et al*., 1998; Hendin *et al*., 1999; Sergerie *et al*., 2007; Moskovtsev *et al*., 2006; Agarwal & Saleh, 2002). In men with varicocele, sperm DNA damage is correlated with abnormal chromatin packaging, abnormal mitochondrial membrane potential (MMP), and apoptosis (Saleh *et al*., 2003; Zini & Libman, 2006; Blumer *et al*., 2008; Sadek *et al*., 2011; Celik *et al*., 2013).

Recently, several studies have reported a relationship between sperm DNA damage and varicocele, but the mechanisms of sperm DNA damage vary among the different studies, and the sample size described may not be sufficient to generate a significant result; therefore, the aim of the present study was to evaluate the effects of varicocele on semen parameters and cytochemical parameters that include sperm DNA fragmentation, chromatin packaging, MMP, and sperm apoptosis.

## MATERIALS AND METHODS

### Population

We carried out a cross-sectional study with semen samples (one per subject), from 2,399 men from couples who attended an infertility clinic. There were two groups: the study group, which was made up of 391 patients with diagnosed varicocele, and the control group, which was made up of 2,008 patients without varicocele. The diagnosis of varicocele was made based on a physical examination. The same physician performed all the exams. All men from the couples who underwent infertility investigation between 2010 and 2015 were included, and the exclusion criteria were azoospermia, any known reproductive tract disease or hormonal therapy, chronic medical disorders, and antioxidant intake. Written informed consent for participation was obtained. The local ethics committee authorized this study.

### Sample collection

Semen samples were collected in sterile containers by masturbation after a mean period of 3.6 days of sexual abstinence (range between 2 to 5 days). A portion of each semen sample was used to analyze the following parameters according to the WHO guidelines (WHO, 2010): volume (ml), pH, concentration (x 10^6^/ml), percentage of spermatozoa with progressive motility (rapid + slow progression), percentage of total sperm motility, number of leukocytes, and percentage of live spermatozoa (vitality). Another portion of each semen sample was immediately processed for morphological analysis, which was performed at high magnification, using motile sperm-organelle morphology examination (MSOME) criteria (Silva *et al*., 2012; Oliveira *et al*., 2014). The remainder of the semen samples was immediately processed for sperm DNA fragmentation analysis using the TdT (terminal deoxyribonucleotidyl transferase)-mediated dUTP nick-end labelling (TUNEL) assay, sperm apoptosis analysis using the Annexin V assay, sperm chromatin packing/underprotamination using chromomycin A3 (CMA_3_) staining, and sperm mitochondrial membrane potential/mitochondrial damage using MitoTracker Green.

### Determination of sperm DNA fragmentation

DNA fragmentation in spermatozoa was measured using the TdT (terminal deoxyribonucleotidyl transferase)-mediated dUTP nick-end labelling (TUNEL) assay as previously described (Franco *et al*., 2008, Oliveira *et al*., 2010; 2014). The number of cells with red fluorescence (TUNEL-positive) was expressed as a percentage of DNA fragmentation.

### Determination of sperm chromatin packaging/underprotamination

Sperm protamine deficiency (underprotamination)/chromatin packaging was measured using chromomycin A_3_ (CMA_3_), as previously described (Franco *et al*., 2012). Two types of staining patterns were identified, namely, bright-yellow fluorescence at the sperm head (CMA_3_ positive/abnormal chromatin packaging - underprotamination) and dull-yellow staining (CMA_3_ negative/normal chromatin packaging - normal protamination). We established the percentages of spermatozoa with abnormal chromatin packaging.

### Determination of sperm mitochondrial membrane potential

Sperm mitochondrial membrane potential (MMP) was determined using MitoTracker Green (MT-G) (Invitrogen - Molecular Probes^®^, Eugene, OR, USA). Live sperm suspensions were incubated in a tube with 20 nm MitoTracker Green (MT-G) for 20 minutes at 37°C in the dark to avoid the breaking down of Molecular Probes^®^. After this period, 0.5 ml of Hoechst 33342 was added into the live sperm suspension and incubated for 10 minutes at 37°C. This probe stains human sperm mitochondria according to MMP; the results are either MT-G positive or MT-G negative. MT-G positive refers to those that have active mitochondria that are colored green in the mid-piece of the sperm. MT-G negative refers to the sperm that did not have labeled mitochondria in the mid-piece. The percentages of spermatozoa with altered MMP/mitochondrial damage (i.e., absence of green fluorescence) were determined.

### Determination of early sperm apoptosis

The Annexin-V binding assay was used for apoptosis analysis. A volume of 20 µl of prepared semen solution containing 1x10^6^ cells/ml was incubated with 100 µl of Annexin-V-Fluos labeling solution (Annexin-Sol) for 15 minutes at room temperature in the dark. The Annexin-Sol consisted of 2 µl of annexin-V-fluos reagent solution (Alexa fluor 488 annexin-v/dead cell apoptosis kit - Invitrogen), 1 µl of propidium iodide (PI), and 1 µl of Hoechst 33342 diluted in 100 µl of incubation buffer. After this period, the mixture (sperm cells and Annexin-Sol) was centrifuged at 600 g for 10 minutes and resuspended in 500 µl of incubation buffer for examination. The percentages of apoptotic cells (defined as the number of spermatozoa that was stained green, and therefore positive for annexin-V, but was not stained red with the propidium iodide dye divided by the total number of spermatozoa) were determined.

### Microscopy evaluation

The quantitative evaluation of all tests (DNA fragmentation, chromatin packaging, apoptosis and MMP) was performed by analyzing at least 200 spermatozoa for each test that were assessed randomly on selected areas on the microscope slides using an Olympus BX50 fluorescence microscope. Texas Red and DAPI filters were used for the TUNEL method, while chromatin packaging, apoptosis and MMP were evaluated using the DAPI filters.

### Quality control

To control for intra-observer and inter-observer variability, multiple fractions of semen samples were obtained from randomly selected patients. Each sample was observed at least three times by each observer (blinded to subject identity). A intra-observer and Inter-observer variation of ≈ 0.5-1% and 0.5-7% respectively were obtained for each parameter analyzed: semen parameters according to the WHO guidelines, normality of the spermatozoon by MSOME, sperm TUNEL-positive, sperm CMA_3_-positive, sperm Annexin V-positive, and sperm MitoTracker Green-positive. These variations are comparable to those of classical sperm-quality parameters (Auger *et al*., 2000).

### Sample size

The sample size was calculated by making a comparison between two proportions. A sample size of 300 subjects in each group had an 80% power to detect an increase of 10% with a significance level of 0.05 (two-tailed).

### Statistical analysis

All variables were initially tested to determine variance homogeneity and data normality. The data is presented as the mean ± standard deviation (SD) or as percentage. A logistic-regression analysis was performed to determine the variables that could be independently associated with sperm DNA damage. In addition, Spearman's correlation was used to verify and identify which cytochemical parameters (sperm packing, early apoptosis or abnormal MMP) are associated with sperm DNA fragmentation in the varicocele group.

The data management and univariate analysis were carried out using StatsDirect statistical software version 2.7.9 software (Cheshire, UK).

## RESULTS

The analysis did not show a correlation between the presence of varicocele and the confounder parameters, including age, BMI, smoking habit, alcohol intake, and abstinence period (Table 1).

**Table 1:** Confounding parameters comparison, including age, BMI, smoking habit, alcohol intake, and abstinence period, between patients with varicocele and men without the pathology.

	Varicocele		Logistic regression		
	Yes (n=391)	No (n=2008)	Odds Ratio	95% Confidence Interval	*p*
Age (years)	37.7±6.3	37.8±6.6	1.00	0.98-1.01	0.97
BMI (kg/m^2^)	28.3±4.4	28.1±19.8	0.99	0.95-1.02	0.35
Smoking habit (%)	10.5	11.9	0.86	0.61-1.23	0.41
Alcohol intake (%)	66.2	67.0	0.97	0.77-1.22	0.77
Abstinence period (days)	3.6±9.1	3.6±2.7	1.00	0.98-1.02	0.84

Statistically significant differences were found between the study and control group with respect to sperm volume, pH, sperm concentration, morphology by MSOME, progressive and total motility, and vitality. There was no statistically significant difference in leukocyte concentration between the groups (Table 2).

**Table 2:** Comparison of the conventional semen parameters and cytochemical analyses between the groups.

Semen parameters	Varicocele		Logistic regression		
	Yes (n=391)	No (n=2008)	Odds Ratio	95% Confidence Interval	*p*
pH	8.1±0.4	8.0±0.4	1.4	1.03-1.82	0.02
Volume (ml)	2.5±1.1	2.8±1.6	0.90	0.82-0.99	0.02
Concentration(x10^6^/ml)	44.4±45.5	76.4±57.2	0.98	0.97-0.99	<0.001
Progressive motility (%)	51.2±17.6	57.0±16.5	0.98	0.97-0.99	<0.001
Total motility (%)	59.1±17.2	63.7±16.0	0.98	0.97-0.99	<0.001
Normal forms (%)	0.4±0.9	0.9±1.5	0.70	0.61-0.81	<0.001
Vitality (%)	60.3±16.3	65.3±15.7	0.98	0.97-0.98	<0.001
Leukocytes (x10^6^/ml)	0.4±1.4	0.7±0.8	1.06	0.96-1.16	0.22
DNA Fragmentation (%)	16.3±8.8	15.3±8.5	1.02	1.01-1.03	0.03
CMA3 positive (%)	58.9±14.8	55.6±15.2	1.02	1.01-1.03	0.001
Abnormal MMP (%)	28.3±16.9	25.4±16.2	1.02	1.01-1.03	0.03
Apoptosis (%)	18.8±7.4	19.2±8.1	0.99	0.97-1.01	0.45

Men with varicocele had a significantly increased percentage of DNA fragmentation, abnormal chromatin packaging (CMA_3_ positive), and abnormal MMP in semen, then men without varicocele. There was no significant difference in early apoptosis between the groups (Table 2).

In the varicocele group, Spearman's analysis observed that early apoptosis and abnormal mitochondrial membrane potential were the two cytochemical parameters that presented a positive and significant correlation with sperm DNA fragmentation. On the other hand, abnormal chromatin packaging and sperm leukocyte concentration (analyzed because of its association with ROS) showed no statistically significant differences in the present study. These results are shown in Table 3.

**Table 3:** Spearman’s correlation within the varicocele group to verify the correlation between cytochemical parameters and sperm DNA fragmentation.

Varicocele group n=391	Spearman’s correlation (%) Sperm DNA fragmentation		
	r	95% Confidence Interval	*p*
Early apoptosis (%)	0.13	0.002 to 0.25	0.04
CMA_3_ positive (%)	0.10	-0.03 to 0.23	0.11
Abnormal MMP (%)	0.32	0.15 to 0.46	<0.001
Leukocytes concentration (x10^6^/ml)	-0.003	-0.11 to 0.10	0.95

## DISCUSSION

Our results demonstrated that patients with varicocele had lower total sperm counts, progressive and total sperm motility, vitality, and normal forms compared to the control group. In addition, these patients had increased percentages of spermatozoa with fragmented DNA. These findings corroborate literature data, which demonstrates that decreased semen quality - determined by the conventional sperm parameters - is associated with clinical varicocele (MacLeod, 1965; Vigil *et al*., 1994; Blumer *et al*., 2008). According to several studies, approximately 25% to 40% of men with semen alterations had varicocele, but the exact mechanism by which this condition impairs sperm quality remains unclear (Hauser *et al*., 2001; Sheehan *et al*., 2014; Pathak *et al*., 2016). It is known that ROS action has deteriorating effects on sperm function, including critical morphological and physiological modifications in both the testes and epididymis, especially in men with varicocele (Gil-Guzman *et al*., 2001).

Despite the availability of different procedures to diagnose varicocele, physical examination, and scrotal ultrasound remain the most commonly used methods. The term clinical varicocele refers to varicoceles detectable on palpation or visual inspection (Nasr Esfahani & Tavalaee, 2012). In the present study, the varicocele diagnosis was made by the same urologist, with the patients in standing position and via scrotal inspection and palpation. The urologist evaluation determined only whether the patient had clinical varicocele, regardless of grade, since the number of men who had varicocele was low. In addition, Bozhedomov *et al*. (2014) didn't find any correlation between the grade of spermatic cord vein dilation and ROS production. The decision for additional exams was at the discretion of the urologist, and too few men were submitted to Doppler evaluation (less than 5% of the total population). Thus, we decided to consider in this study only the clinical examination as a criterion for the diagnosis of varicocele.

Data in the literature have demonstrated an increase in DNA fragmentation in men with varicocele and a clear association between oxidative stress and worse sperm parameters, including conventional parameters and sperm DNA damage (Bungum *et al*., 2007; Razi *et al*., 2011; Nasr Esfahani & Tavalaee, 2012). This data and the outcomes of this study have demonstrated that there is an increase in DNA fragmentation in the sperm from the varicocele group compared to the control group.

Proper function and protection of sperm DNA depend on the remolded and condensed chromatin structure that is associated with an exchange of histone by protamine in the spermatid nucleus during the later stages of spermatogenesis -which is a crucial step in male fertility (Ward & Coffey, 1991; Steger *et al*., 2000). This process is responsible for the compaction and stabilization of the sperm nucleus, and therefore, protects the sperm genome from oxidative stress, toxic metabolites, and excessive heat; a defect in this process makes the spermatozoa more susceptible to DNA damage, which is consequently associated with male infertility (Kosower *et al*., 1992; Aitken & Krausz, 2001; Smith *et al*., 2006; Zini & Libman, 2006). There is clinical evidence to show that abnormal sperm chromatin packaging is correlated with the reduced ability of spermatozoa to fertilize oocytes in normal conception or during ART procedures (Sakkas *et al*., 1996; Filatov *et al*., 1999; Talebi *et al*., 2008). In accordance with other authors that have used (CMA_3_) to evaluate the DNA chromatin status in men with varicocele (Nasr-Esfahani *et al*., 2001; Singleton *et al*., 2007; Talebi *et al*., 2008), this study has demonstrated an increase in the levels of abnormal chromatin packaging in the semen from patients with varicocele. Other authors, using different methods, have also verified the chromatin packaging quality in men with varicocele, and similar results have been found (Salsabili *et al*., 2006; Sadek *et al*., 2011).

Mitochondria are important organelles that are present in several biologic processes, including spermatogenesis. Sperm motility depends on the normal function of the mitochondria that are in the mid-piece of the spermatozoa where ATP is produced, and it has been demonstrated that mitochondrial dysfunction is associated with reduced sperm functionality and male infertility (Amann, 1989; O'Connell *et al*., 2002; St John *et al*., 2005). Mitochondria are the major sources of intracellular ROS, and controlled ROS levels are needed for proper sperm function, including capacitation, the acrosome reaction, hyperactivation and fertilization (Kothari *et al*., 2010; Aitken *et al*., 2012). It is important to note that abnormal mitochondrial activity is related to increased levels of ROS, which may lead to decreased sperm motility and sperm DNA damage (Koppers *et al*., 2008; Sharma *et al*., 2013). Varicocele has been correlated with a high percentage of sperm with inactive mitochondria, and the oxidative stress found in patients with this condition may be a plausible explanation for this observation (Blumer *et al*., 2008; Koppers *et al*., 2008; Sharma *et al*., 2013). In our study, we observed that men with varicocele present with increased levels of abnormal mitochondrial membrane potential and therefore increased levels of inactive mitochondria, which is in accordance with literature data.

Germ cells die during normal spermatogenesis, by a process known as apoptosis, and is estimated to be responsible for the loss of up 75% of potential spermatozoa (Rodriguez *et al*., 1997; Sakkas *et al*., 2003; Said *et al*., 2004). Apoptosis is one of the main pathophysiological mechanisms of testicular dysfunction in men with varicocele, and there are three possible causes for apoptosis: higher testicular temperature, reduced levels of testicular testosterone, and the buildup of toxic metabolites in the semen (Sakkas *et al*., 2003; Benoff *et al*., 2004; Lee *et al*., 2009). In addition to early apoptosis, increased necrosis and sperm degeneration are also observed in patients with varicocele, probably because these processes are facilitated in these patients (Benoff *et al.*, 2004, Nasr Esfahani & Tavalaee, 2012). The increased molecular levels of ROS that result from varicocele would directly cause sperm DNA damage, and this has been demonstrated to be one of the most common ways to induce apoptosis (Simşek *et al*., 1998; Hendin *et al*., 1999; Cam *et al*., 2004). Consistent with the literature (Simşek *et al*., 1998; Hendin *et al*., 1999; Cam *et al*., 2004; Chen *et al*., 2004), this study has shown an increase in early apoptosis levels in patients with varicocele compared to the group of men without venous vascular impairment of the testes.

Possible sources of ROS are not entirely known, but spermatozoa and leukocytes have been demonstrated to be involved in the production of ROS at levels that are considered physiological (Cho *et al*., 2016). Abnormal spermatozoa and leukocytes, especially when activated by infection or inflammation, are potential sources of excessive ROS production and the consequent oxidative stress in the semen of patients with varicocele (Ochsendorf, 1999; Gil-Guzman *et al*., 2001). However, our study shows that leukocytes may not be involved in an extrinsic pathway of ROS production and do not appear to be a determinant in causing sperm DNA damage.

In conclusion, this study has shown a correlation between the presence of varicocele and a decrease in semen quality. In addition, the presence of this pathology increases DNA fragmentation, abnormal chromatin packaging, and abnormal mitochondrial activity levels. In men with varicocele, high levels of sperm mitochondrial inactivity and sperm apoptosis are associated with increased levels of sperm DNA fragmentation; however, DNA damage is not associated with sperm chromatin packaging and sperm leukocyte concentration. In patients with clinical varicocele, antioxidant intake and varicocele repairs should be considered as a feasible way to reduce sperm DNA damage. However, well-designed studies are required to demonstrate, in details, how varicocele impairs fertility.
